# Non-coding antisense transcription detected by conventional and single-stranded cDNA microarray

**DOI:** 10.1186/1471-2164-8-295

**Published:** 2007-08-29

**Authors:** Johan Vallon-Christersson, Johan Staaf, Anders Kvist, Patrik Medstrand, Åke Borg, Carlos Rovira

**Affiliations:** 1 Department of Oncology, Institute of Clinical Sciences, and SWEGENE DNA microarray resource center, Lund University, Barngatan 2:1, SE-221 85 Lund, Sweden; 2 Genomics and Bioinformatics, Department of Experimental Medical Science, BMC C13, SE-221 84 Lund, Sweden; 3 Lund Stem Cell Centre, University of Lund, BMC C13 SE-221 84 Lund, Sweden

## Abstract

**Background:**

Recent studies revealed that many mammalian protein-coding genes also transcribe their complementary strands. This phenomenon raises questions regarding the validity of data obtained from double-stranded cDNA microarrays since hybridization to both strands may occur. Here, we wanted to analyze experimentally the incidence of antisense transcription in human cells and to estimate their influence on protein coding expression patterns obtained by double-stranded microarrays. Therefore, we profiled transcription of sense and antisense independently by using strand-specific cDNA microarrays.

**Results:**

Up to 88% of expressed protein coding loci displayed concurrent expression from the complementary strand. Antisense transcription is cell specific and showed a strong tendency to be positively correlated to the expression of the sense counterparts. Even if their expression is wide-spread, detected antisense signals seem to have a limited distorting effect on sense profiles obtained with double-stranded probes.

**Conclusion:**

Antisense transcription in humans can be far more common than previously estimated. However, it has limited influence on expression profiles obtained with conventional cDNA probes. This can be explained by a biological phenomena and a bias of the technique: a) a co-ordinate sense and antisense expression variation and b) a bias for sense-hybridization to occur with more efficiency, presumably due to variable exonic overlap between antisense transcripts.

## Background

Non-coding RNAs have recently been reported as more common, more diverse, and accredited more important functions than previously anticipated [1-3]. Among the most abundant non-coding transcripts, there is a group called natural antisense transcripts (*NATs*) that carries regions of perfect complementarity to protein coding (sense) RNAs [4-7]. *In silico *studies of available transcript sequence data have found that up to 24% of human protein coding loci also encode cis-*NAT*s [8,9]. However, antisense transcripts tend to be poly(A) negative and nuclear localized [10]. If this is true, the abundance of *NAT*s (*cis *and *trans*) may be higher yet, since nuclear non-polyadenylated transcripts are underrepresented in transcript sequence databases.

This fact may have important implications for researchers, not only because of their potential biological function but they may also turn out to be influential on the interpretation of large experimental data sets. For instance, the cDNA microarray technique has been used in genome-wide expression studies to address basic questions about gene function and in the pursuit of a more precise molecular classification of tumors. In this case, the ability to monitor the expression of thousands of genes simultaneously has allowed the identification of disease-specific subsets of genes useful to improve diagnosis and disease management [11]. The majority of the more than 90.000 microarray expression profiles released through NCBI was obtained with double stranded cDNA capture probes and is assumed to reflect the pure expression of the sense transcripts used as templates for cDNA synthesis. However, the widespread expression of natural antisense transcripts (*NAT*s) invalidates this assumption since double-stranded probes will show the combined expression of both the intended sense target and any *NAT *with complementary sequence [12,13]. Still, for nine out of ten cases, signals from double-stranded cDNA probes correlates with those obtained from sense specific oligonucleotide platforms [14]. Based on these observations, we reasoned that antisense transcripts are either not efficiently detected by conventional cDNA capture probes or that important information must be hidden behind this paradox.

Therefore, we modeled a typical cDNA microarray tumor-classification analysis and compared the results from conventional double-stranded cDNA capture probes with single stranded cDNA capture probes capable of monitoring opposite strands of each cDNA independently. We detected a number of antisense signals that exceed by far the number of known antisense transcripts. The detected signals showed a clear cell specific expression pattern with a common core group of antisenses expressed in all analyzed materials. Moreover, antisense transcripts displayed a prevalent tendency to be positively correlated with the expression of their corresponding sense counterparts. This confirms the idea that a large part of the data obtained from conventional double-stranded cDNA microarrays are in fact compounded signals product of both sense and antisense hybridization. Yet, detection of antisense transcription by conventional double-stranded cDNA microarrays does not strongly distort the relationship between expression profiles of the analyzed samples compared with those obtained from pure sense signals. This is most likely due to the observed coordinate regulation of senses and antisenses and a more efficient hybridization of sense strands because a different exon structure of antisense transcripts and the sense transcripts used for cDNA synthesis.

## Results and discussion

### Production of single-stranded microarrays

**Figure 1:**
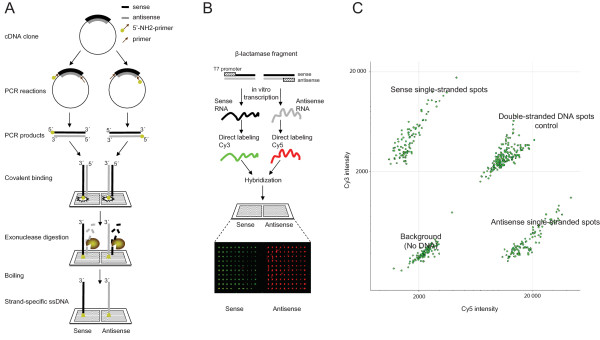
***In situ *single stranded cDNA microarray production method (a) **Strand-specific end modifications (amino-linkers) are incorporated into DNA by two parallel PCR reactions. After clean-up, the PCR product for each end-modified strand is printed separately onto the same microarray glass slide. The amino terminal groups are further coupled to the glass. The probes are digested with 5'-3' T7 gene 6 exonuclease and then immersed in boiling water. As a result of this treatment, only end-modifed strands remain attached to the surface. **(b) **Hybridization of labelled *in vitro *transcribed RNA from the β-lactamase gene with β-lactamase single-stranded sense and antisense DNA capture probes. Control spots containing ds-DNA probes or amino-modified PCR primers alone were also included on the array (not shown). Both probes and controls were spotted in 10 × 10 replicates. **(c) **Scatter plot showing the distribution of raw median pixel signal intensities from the hybridization performed in (b). Signals from all replicates are clearly discriminated according to the strand from which they originated (sense or antisense). Control spots for background intensities (printing buffer, PCR and amplification primers) demonstrate the specificity of the single stranded probes.

We generated strand specific cDNA probes *in situ *after covalently binding NH_2_-modified cDNA inserts onto cross-linked N-hydroxysuccinamide slides in a strand specific manner. Specific binding of 5' DNA ends serves two different but additive purposes. First, 5' end-specific binding provide protection against *in situ *enzymatic attack of highly processive 5'-3' exonucleases; unbound strands could then be exposed to enzymatic degradation. Second, only 5'-end modified strands will be covalently bound, rendering non-modified strands vulnerable to easy removal by heat denaturation. We found that the most reliable method for processing double stranded cDNAs into single stranded capture probes was the sequential application of both approaches. The procedure is schematically depicted in Figure 1a. To validate the method, microarrays containing single-stranded sense and antisense probes and double-stranded probes (PCR products NH_2_-modifed at both 5'ends that remain double-stranded after processing) were generated from a 1 kb fragment containing the β-lactamase gene. Hybridizations were performed with equimolar amounts of Cy3- or Cy5- direct-labelled sense and antisense β-lactamase transcripts (Figure 1b). Sense probes showed strong signal from Cy3 labelled sense cDNA (532 nm wavelength) and signal equivalent to background level in the Cy5 channel (635 nm wavelength). Contrary, antisense probes detected strong signal from Cy5-labeled antisense cDNA while Cy3 signal remained equivalent to background (Figure 1c). We conclude that our method can produce selective single stranded DNA capture probes from double-stranded PCR products *in situ *with an efficacy and specificity sufficient to eliminate detectable levels of complementary strand, thus producing a selective array of strand-specific capture probes.

### Strand-specific cDNA microarrays

**Figure 2:**
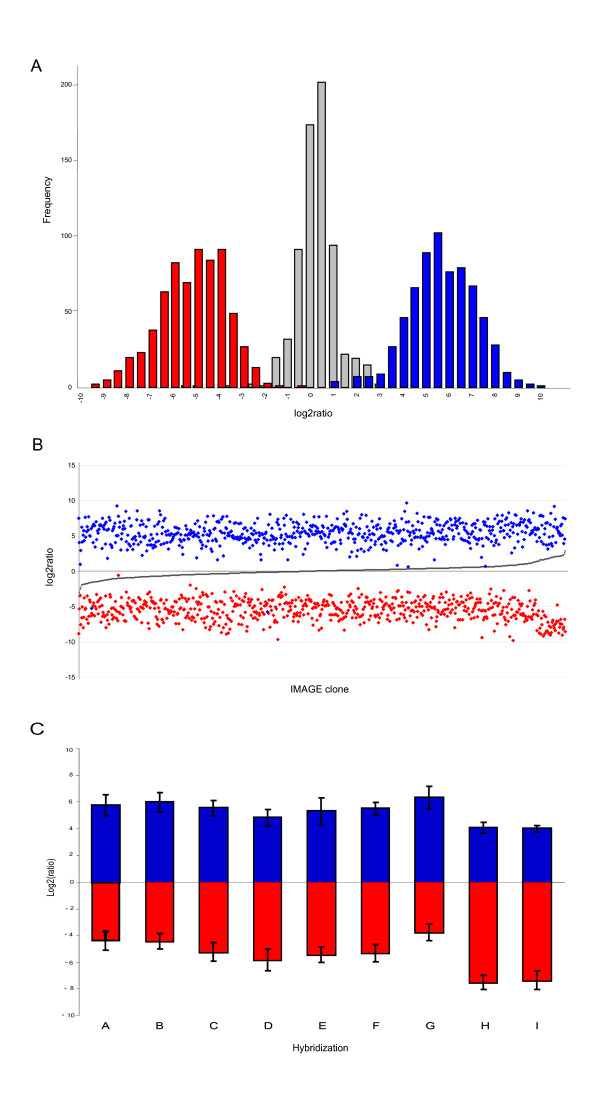
**Strand specificity of *in situ *produced sense/antisense cDNA microarray features. (a) **Histogram showing the distribution of log_2 _ratios after hybridization to single stranded C (blue), N (red), and double stranded CN (gray) capture probes with labelled Cy3- M13 forward and Cy5 – reverse sequencing primers (The log_2 _ratios for C and N probes from cDNA clones of plate IRAT3 were centered around -5 and 5 respectively, as expected since their cloning sites were reversed compared to the other clones, but to simplify the presentation the values are inverted). **(b) **log_2 _ratios for probes shown in panel matched on cDNA clone ID and sorted on ascending log_2 _ratio for the respective CN probe. **(c) **Mean log2ratios for β-lactamase spike-in sense (blue) and antisense (red) control features for hybridizations A to I. For individual features, log_2 _ratios were calculated using untransformed background corrected data (Median FG – Median BG). For mean log_2 _ratio calculations, only features where MedianFG > MedianBG in both channels, i.e. Cy3 and Cy5, and where SNR > = 3 in either channel, were included. Error bars denote +/-1SD. Mean log2ratio were calculated using values from between 66 and 70 replicated control spots.

Following this test, we prepared strand-specific sense and antisense cDNA probes, and double-stranded probes, from 960 randomly selected full-length cDNAs from the MGC clone collection. Both strand specifically 5' NH_2_-modified PCR products for sense and antisense probe generation and NH_2_-modified products at both 5' ends were printed on the same surface. In the following, sense probes are termed C for coding and antisense probes N for non-coding. Double-stranded probes are termed CN. To verify the specificity of our cDNA capture probes, we hybridized the arrays with Cy3 or Cy5 labeled universal primers used to amplify all cDNA inserts (see Methods). Data from the test hybridization shows that CN probes produce log_2 _ratio intensities centered on 0, demonstrating that they captured both labeled targets. Conversely, strand specific C and N probes produced log_2 _intensity ratios centered approximately on 5 and -5 respectively (Figure 2a). Figure 2b show log_2 _ratios for C, N, and CN probe for each cDNA clone sorted on ascending basis for respective CN probes. With very few exceptions, each probe set consistently produced strand specific probes equally well across all cDNAs.

### Antisense detection of N probes hybridized with total RNAs

Next, we modeled an experimental design typically used for tumor classification studies. We performed hybridizations using direct-labeled randomly primed total RNA extracted from eight breast-cancer cell lines and one derived from normal breast epithelium against Universal Human Reference RNA as a common reference following standard protocols (see Methods). To control for even processing into strand-specific probes we printed internal β-lactamase DNA control spots in each block and included β-lactamase spike-in sense and antisense control targets in either samples or reference. Their hybridization signals confirmed that detected N signals reflect hybridization to the N probe over the entire array (Figure 2c).

**Figure 3:**
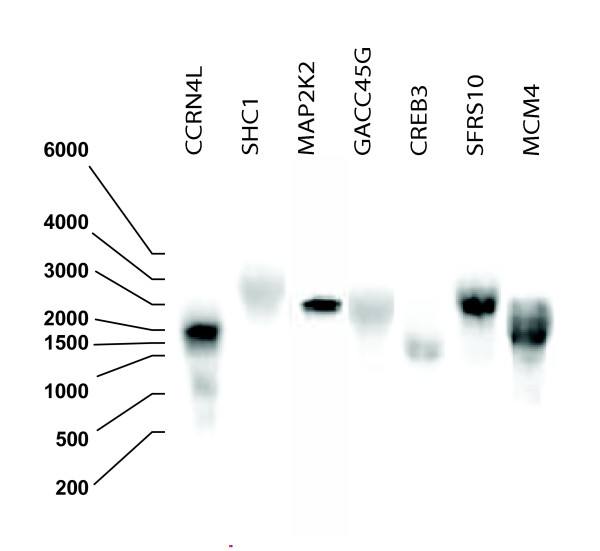
**Validation of putative antisense RNA transcripts**. Northern blot hybridizations of total RNA with sense P^32^-labeled riboprobes corresponding to a sample set for which antisense RNAs were detected on the single-stranded microarray but had not preciously been reported. cDNAs correspond to: ubiquitin C partial cds CCRN4L (BC000449), src homology 2 domain containing transforming protein SHC1 (BC014158), mitogen activated protein kinase 2 MAP2K2 (BE543096), growth arrest and DNA-damage inducible protein gamma GACC45G (BC000465), c-AMP responsive element binding protein 3 CREB3 (BC010158), splicing factor SFRS10 (BC000451) and MCM4 minichromosome maintenance deficient 4 MCM4 (BC036035).

We initially analyzed the detection capacity of N probes. We applied a stringent filter criterion including signals with signal-to-noise (SNR) ratios above 10. Across all nine hybridizations, we detected 776 N signals in the cell lines and 885 N signals in the reference, out of the set of 960 probes (Table 1). We filtered for non-specific cross-hybridization with repetitive elements in the probe sequence [19], extended self-complementarity between the C and N probes, and C and N probes with sequence complementarity to the same transcript. We identified repeat sequences of 40 or more continuous bases in 358 of the 960 cDNA used for probe synthesis, 11 cases with extended self-complementarity between C and N probes and 87 cases for which the C and N probes matched the same RefSeq mRNA [20]. After filtering for potentially spurious signals, 470 N signals from the cell lines and 556 from the reference were interpreted as detecting putative antisense transcripts. The N signals detected in the cell lines vary from 27% (UACC812) to 74% (CAMA-1) of the cDNAs that also showed sense transcription. We compared these signals with *NAT*s identified by the most comprehensive study published [9]. Despite significant variations in the number of N signals, the percentage of detected reported *NAT*s remains unchanged in each cell line (22.2% on average, SD 1.2). In the reference channel, an average of 87.79% cDNAs detecting C signals also produced N signals (SD 5.65). As many as 116 putative antisense transcripts in the cell lines and 398 in the reference were detected in all nine hybridizations and, again, a similar proportion of reported antisense transcripts in each group were noted (21.6% respectively 24.1%). We observe greater variation in the number of detected N signals for the cell lines compared to the reference, indicating that biological variation is larger than variation resulting from technical replication of measurements (Table 1). Here, we aimed to gain insight into the occurrence of *NAT*s and to analyze their influence in the context of microarray hybridizations rather than to rigorously screen for novel antisense transcripts. However, N signals show biological variation that tend to be sample specific, yet reveal a common core detected in all experiments. This group includes genes involved in apopotosis and programmed cell death. If a significant proportion of these detected unannotated transcripts were random artifacts, we would expect the common core to show a lower proportion of known annotated transcripts. Nevertheless, it is remarkable that the proportion of annotated antisense transcripts for the common core is similar to that estimated for each cell line separately (21.6% compared to 22.2% on average for the cell lines). To verify the nature of the detected unannotated transcripts we radio-labeled sense strands from ten randomly selected cDNAs by *in vitro *transcription and used them as hybridization riboprobes in Northern blot hybridizations. Seven of the ten tested riboprobes were able to identify complementary transcripts in the total RNA mixture (Fig. 3). These observations strengthen the view that most detected N signals might represent true *NAT*s (either *cis *or *trans*). This impression is reinforced by the fact that hybridization with tilling arrays predicted that only one tenth of the total number of transcription units is known in human [21] and the observed well-defined expression pattern of N signals described subsequently. We find no reasons to conceive that this extended antisense transcriptional activity is associated with the nature of the analyzed materials (mostly cancer cell lines) as MCF-10, derived from normal breast epithelia detected a number of antisense signals second closet to average. In summary, as much as 88% of the tested conventional cDNA probes could potentially produce microarray signals reflecting differences in sense or antisense expression, or both, in one sample or both of sample and reference.

**Table 1:** Hybridization signals detected in sample channel and reference channel

	Detected N signals		Masked IMAGE ID					Putative antisense signals			C signals	
				
	total*	reported**	repeats***	double hits****	self-complementarity^+^	repeats, double, or self^++^	reported^+++ ^	total^++++^	reported^$^	new^$$^	total^$$$^	% paired N signal^$$$$^
UACC812 (A)	281	59	148	61	7	151	31	**130**	**28 **(21,5%)	**102**	353	27
ZR7530 (B)	316	67	149	57	8	153	33	**163**	**34 **(20,9%)	**129**	353	35
BT474 (C)	610	134	252	74	11	257	50	**353**	**84 **(23,8%)	**269**	478	64
MDA361 (D)	603	132	241	71	0	246	49	**357**	**83 **(23,2%)	**274**	465	66
MCF10A (E)	556	114	241	72	11	246	46	**310**	**68 **(21,9%)	**242**	473	57
SKBR3 (F)	449	94	192	65	8	196	41	**253**	**53 **(20,9%)	**200**	431	48
UACC893 (G)	516	110	216	67	8	220	43	**296**	**67 **(22,6%)	**229**	442	56
MDA453 (H)	562	115	233	72	9	238	47	**324**	**68 **(21,0%)	**256**	460	60
CAMA1 (I)	695	151	276	76	11	282	53	**413**	**98 **(23,7%)	**315**	501	74

Mean	509,78	108,44	216,44	68,33	9,22	221	43,67	288,8	64,78 (22,2%)	224	439,56	54,14
SD	137,63	30,55	44,89	6,32	1,56	45,7	7,53	92,37	23,10 (1,2%)	69,52	53,03	15,18
Nmbr(union)	776	169	300	78	11	306	60	470	109 (23,2%)	361	511	
Nmbr(intersection)	244	52	125	55	7	128	27	116	25 (21,6%)	91	315	
												
Reference (A)	753	169	296	78	11	303	62	**450**	**107 **(23,8%)	**343**	510	81
Reference (B)	751	169	288	79	11	295	59	**456**	**110 **(24,1%)	**346**	505	82
Reference (C)	813	185	303	77	11	309	65	**504**	**120 **(23,8%)	**384**	513	90
Reference (D)	808	179	301	79	11	308	62	**500**	**117 **(23,4%)	**383**	514	89
Reference (E)	806	178	301	80	11	307	62	**499**	**116 **(23,2%)	**383**	513	89
Reference (F)	826	186	307	79	11	314	65	**512**	**121 **(23,6%)	**391**	519	91
Reference (G)	745	169	288	78	11	294	62	**451**	**107 **(23,7%)	**344**	502	80
Reference (H)	822	182	311	78	11	317	66	**505**	**116 **(23,0%)	**389**	520	90
Reference (I)	874	196	316	79	11	323	64	**551**	**132 **(24,0%)	**419**	524	97

Mean	799,78	179,22	301,22	78,56	11	307,8	63	492	116,22 (23,6%)	375,8	513,33	87,79
SD	42,63	9,24	9,54	0,88	0	9,58	2,18	33,63	7,84 (0,4%)	26,02	7,07	5,65
Nmbr(union)	885	198	322	81	11	329	66	556	132 (23,7%)	424	525	
Nmbr(intersection)	673	153	269	75	11	275	57	398	96 (24,1%)	302	489	

*Number of IMAGE clones where N probe SNR > 10

**Number of IMAGE clones previously reported (ref 9) as transcribed from antisense (out of Detected N signals total*)

***Number of IMAGE clones with > = 40 continuous bases homologous to repeats (out of Detected N signals total*)

****Number of IMAGE clones with > = 30 continuous bases on both sense- and antisense strand that match RefSeq (out of Detected N signals total*)

^+^Number of IMAGE clones with > = 30 continuous bases complementary between sense- and antisense transcript (out of Detected N signals total*)

^++^Number of IMAGE clones with either repeats***, double hits****, or self-complementarity^+^(out of Detected N signals total*)

^+++^Number of IMAGE clones that overlap between masked++and clones priviously reported** (out of Detected N signals total*)

^++++^Number of IMAGE clones with putative antisense transcription, i.e. those clones that are not masked^++^(out of Detected N signals total*)

^$^Number of IMAGE clones previously reported as transcribed from antisense out of, Putative antisense signals total^++++^(percentage within parenthesis)

^$$^Number of IMAGE clones not masked^++ ^and not privously reported** as transcribed from antisense (out of Detected N signals total*)

^$$$^Number of IMAGE clones with putative sense transcription, i.e. where C probe SNR > 10, excluding clones that are masked^++^

^$$$$^Percent of IMAGE clones displaying N signal out of those that display C signals excluding clones that are masked^++^

Note: values are paired per clone, i.e. the number of IMAGE clones with C signal and N signal as percentage of the number of IMAGE clones with C signal

### Relationship between C and N signals

**Figure 4:**
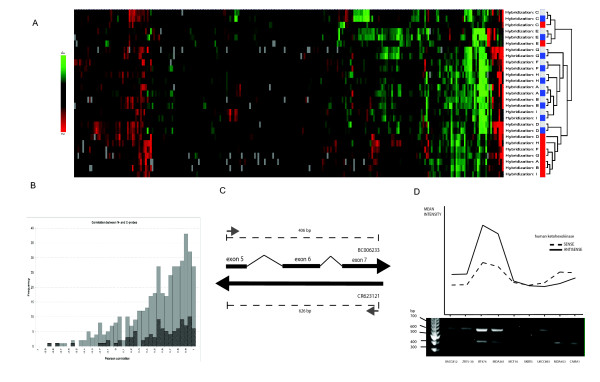
**Sense and antisense expression profiles**. **(a) **Unsupervised cluster analysis of expression profiles detected by C (blue), N (red) and CN (grey) capture probes across 9 cell lines. A: UACC812; B: ZR75-30; C: BT474; D: MDA361; E MCF10A, F: SKBR3; G: UACC893; H: MDA453 and I: CAMA1. Columns in the gene expression matrix correspond to IMAGE clone IDs. Each cell line is represented by one row. Minimum presence per cDNA clone was set to 7 out of 9 in each data set. **(b) **Correlation between N- and C-probes across hybridizations A to I for 430 probe pairs, i.e. IMAGE clones. Frequency of Pearson correlation is shown as bars with a bin width of 0.05 (gray bars). For each frequency, the contribution of probe pairs from IMAGE clones previously reported as antisense transcribed is shown as striped areas. Correlation calculations are performed on log2ratios using within hybridization mean log2ratio per reporter across features with SNR > = 10 in either channel, i.e. Cy3 or Cy5. IMAGE clones were removed if their respective N- and C- probes were suspected to potentially cross-hybridize or if their number of missing values across hybridizations was > 2 for either N- or C- probe. **(c) **Schematic representation of the overlap between KHK (BC006233) and CR623121 transcripts and rational of the RT-PCR reaction aimed to verify the observed coordinated regulation of sense and antisense transcripts. Black arrows represent orientation of transcription for each RNA. Grey arrows mark positions for the PCR primers used in (d). Distance between primers positions refer to the final amplification products. **(d) **Relative expression of KHK and its antisense CR623121 detected by RT-PCR across all cell lines. Two amplicons of 406 and 626 bp corresponding to KHK sense and antisense transcripts were obtained. Lower panel: ethidium bromide staining after separation in 1% agarose. Band intensities were quantified with the Kodak 1D Image Analysis Software and mean intensities were plotted.

To analyze closely antisense expression and to explore the extent to which antisense signals might influence protein-coding profiles detected by double-stranded microarrays, we jointly investigated the relationship between signal from C, N, and CN probes. First, we visualized distances between expression profiles derived from separate probes using unsupervised cluster analysis. We regarded expression data from different probes as individual data sets, separating hybridizations into one C, one N, and one CN profile matched on clone IDs. Cluster analysis using probes derived from 260 cDNA clones show that C and CN profiles co-segregate within each cell line (Figure 4a). The majority of N profiles form a cohesive cluster well separated from C and CN profiles for the same group of cell lines. For two cell lines, the N, C and CN profiles separate from all other cell lines but within each group the C and CN profiles co-segregate with the N profile clearly separated. Visual inspection revealed that C and N signals seem to follow coordinated variation. To corroborate this observation we calculated pair wise correlation between C signal and N signal across the nine hybridizations for corresponding cDNA clones. Figure 4b shows the distribution of Pearson correlations for 430 C and N signal pairs, evidencing a tendency for positive correlation between them. The same coordinated pattern was apparent when only probe pairs from cDNA clones with previously known *NAT*s were considered. Overall, only a minority of clones showed an inverse relationship between senses and antisense expression levels. These probes seem to be responsible for the separation between C and N profiles. A tendency for positively correlated co-expression of sense and antisense transcripts has been observed in large-scale studies performed in mouse [22]. However, studies on human material claimed that expression of members of sense-antisense pairs follow an inverse relation which was supposed to agree with a mechanistic model according to which increasing expression of one of the members downregulates transcription of the other [23]. Our results contradict this observation. To verify our observation by an alternative method, we selected the gene encoding human ketohexokinase as representative example (KHK, accession number BC006233). In our microarray measurements, KHK's transcription displayed a high positive correlation between N and C signals (Pearson's correlation coefficient *r *= 0,97). KHK exons 5, 6 and 7 fully overlap clone CR623121 which transcription proceeds in the antisense orientation. We designed a forward primer on KHK exon 5 and reverse primer on exon 7 targeting the regions of sequence complementarity in both cases. Due to different splicing, this primer pair would allow simultaneous amplification of a 406 bp KHK fragment and a 626 bp product from the CR623121 transcript (Figure 4c). Figure 4d shows the outcome of these RT-PCR experiments. A tight coordinated variation between expression levels of KHK and its antisense transcript was obtained across all tested cell lines (*r *= 0,80) confirming our microarray results. Thus, the analyzed genes appear to follow a pattern of more or less coordinated up- or down-regulation of both strands in parallel. Supported on previously reported data [24], we interpret the coordinated expression of N with C signals as evidence for tight sense-antisense regulation rather than the effect of a stochastic transcriptional noise. Moreover, if sense/antisense expressions tend to be correlated for protein-coding loci, we speculated that profiles should not substantially differ, regardless of whether the array probes are single- or double-stranded.

### Single-stranded compared to conventional double stranded cDNA arrays

**Figure 5:**
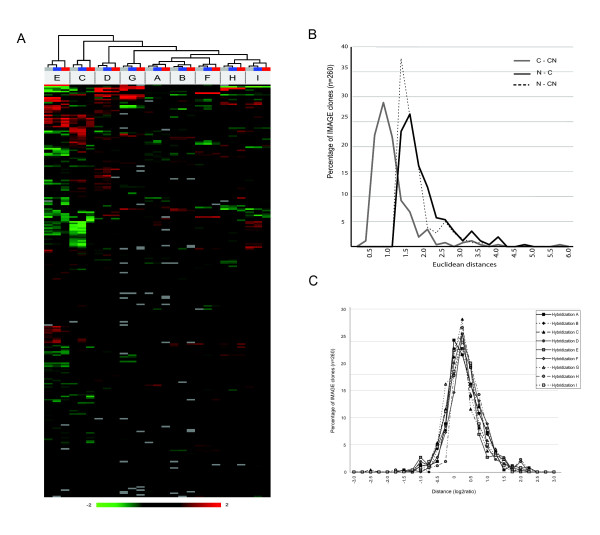
**(a) **Cluster analysis of C (blue), N (red) and CN (grey) expression profiles where all samples were median-centred and treated as they were independent experiments. Minimum presence per cDNA clone was set to 7 out of 9 in each data set. **(b) **Histogram of pair-wise Euclidean distances over all samples for IMAGE clones. For each IMAGE in 9 dimensions, i.e. hybridization 1–9, the Euclidean distance between C and N is √(Σi = 1 (C_i_-N_i_)^2^) where C_i _and N_i _is the log2ratio for C and N probe respectively for hybridization i. **(c) **Histogram of distances between C and N probes for all IMAGE for each hybridization.

To investigate further the connection between C and CN profiles we calculated pairwise Euclidean distances between C, N and CN signals across cell lines. Taken together, these data indicate that C and CN profiles are more similar across the hybridized samples than either is to N profiles (Figure 5b). To exclude the possibility that a small number of outliers caused the difference, we investigated the distribution of N to C distance per cDNA clone within hybridizations (5c). We found a very similar distribution for each hybridization suggesting that antisense transcription – while it is genome-wide – does not significantly distance profiles obtained from pure sense probes from those derived from dsDNA.

To test this conjecture, we treated C, N and CN profiles from each cell line as part of an independent experiment. Expression data from each probe set were median-centered separately and their expression profiles compared by cluster analysis (Figure 5a). All cell lines segregate in a similar manner, independent of the nature of the probe. Moreover, the relationship between expression patterns detected by C, N and CN capture probes for each one of the nine samples was similar for all of them. C and CN segregated consistently close to each other leaving N probes as outliers. We observed a closer distance between C and CN expression than between either of these profiles and N, across all performed experiments. This could be explained by the fact that N probes are derived from sense transcripts cDNAs and are not a mirror copy of *NAT*s. Therefore, mature antisense transcripts and N probes would not be perfectly complementary but, assuming that all sense and antisense immature transcripts undergo splicing, will display restricted regions of exon overlap. This situation should favor the hybridization dynamics of C probes since renaturation kinetic is directly dependent on the length of the complementarity with the immobilized probes [25].

## Conclusion

In spite of stringent signal-to-noise ratio criteria, our strand-specific cDNA microarrays detected a number of antisense transcripts that exceeds by far the number of previously annotated antisense genes in humans. Partial validation and observed well defined expression patterns suggest that a considerable fraction of these signals might represent *bona-fide *unannotated antisense transcripts (both *cis*- and *trans*). These *NAT*s are expressed in a cell specific manner and displayed a strong tendency to follow the expression pattern of their sense counterparts.

Since antisense transcription data is embedded in double stranded cDNA array experiments, it is expected to affect signals and gene clusters, and would make data validation difficult of array results by other means. We analyzed this issue and found that even if antisense transcription is genome-wide it exerts a restricted influence on the interpretation of conventional cDNA microarray data. Today, these problems can be circumvented in well-annotated genomes because strand specific expression can be discriminated by the use of oligonucleotide capture probes. However, strand-specific oligo design would be hampered today by the limited access to antisense sequence data, as shown here. Although reassuring the overall validity of cDNA microarrays in previous tumour-classification studies, our results emphasize the need for further development of methods that accurately measure strand-specific expression.

## Methods

### Preparation of capture probes

To produce cDNA arrays, 960 full-length cDNA clone inserts from the MGC collection [15] (plates IRAT3, IRAT33, IRAU2, IRAU19, IRAU31, IRAU44, IRAU46, IRAU62, IRAU68 and IRAU71) were amplified three times using different combinations of 5'-amino modified M13 universal sequencing primers (GTTGTAAAACGACGGCCAGTG forward and CACACAGGAAACAGCTATG reverse). One reaction set used 5'-amino C-6 link modified M13 forward paired with a non-modified reverse primer. A second reaction used 5'-amino C-6 link modified M13 reverse primer paired with a non-modified forward primer. A third reaction used 5'-amino C-6 link modified primers in both orientations to produce double-stranded probes. Amino-modified M13 forward primers were used to produce sense (coding) strand probes and M13 reverse primers to produce non-coding antisense strand probes in all reactions except those corresponding to plate IRAU3 (cloned into pCMV-SPORT6 with the cloning site in opposite orientation). To produce capture probes for the test array used in Figure 1, a one kilo-base (kb) fragment from the β-lactamase gene was amplified using the same procedure but with plasmid DNA as template and primers GGCACCTATCTCAGCGATCT and GCGGAACCCCTATTTGTTTA.

After amplification, all PCR products were precipitated with ethanol and resuspended in phosphate buffer at approximately 100 ng/μl for further deposition on arrays. Triplicates of sense and antisense amino-modified β-lactamase PCR products were also deposited on each of the blocks of the cDNA array to monitor the processing of double-stranded DNA to single-stranded (ssDNA).

Amino-modified PCR products were spotted onto CodeLink activated slides (Amersham) using a MicroGrid II array robot (BioRobotics). Capture probes on slides used in Figure 1 were printed as 10 × 10 replicates. The test slides also included background control spots produced by processing PCR reactions without template (PCR primers with no amplification product).

After printing, slides were coupled in a saturated NaCl chamber overnight at room temperature, blocked in 50 mM ethanolamine, 0,1 MTris (pH 9) at 50°C 30 min followed by washes in distilled water and 4 × SSC/0.1% SDS at 50°C. The deposited dsDNA was subsequently digested *in situ *with T7 exonucelase 6. Slides were overlaid with 45 μl exonuclease reaction mixture (1 U/μl T7 exonuclease 6 in 1× reaction buffer, New England Biolabs), covered with a coverslip and left to incubate for 30 minutes at 25°C in a CMT Corning hybridization chamber. Following *in situ *digestion, probes were denatured by immersing the arrays in boiling water for two minutes.

### Microarray hybridization

To produce targets for hybridization of the test array shown in Figure 1, the T7 RNA polymerase promoter was incorporated by PCR in the sense or antisense orientation relative to the β-lactamase gene, and corresponding transcripts were synthesized by *in vitro *transcription. For the cDNA arrays, total RNAs were extracted from cell lines ZR-75-30, BT-474, SKBR-3, MDA-361, UACC-812, UACC-893, CAMA1, MDA453 and MCF-10 with Trizol (Invitrogen), purified with Qiaex (Qiagen), and integrity was checked on a Bioanalyzer (Agilent). Targets of *in vitro *transcribed β-lactamase, total RNA from cell lines, or Human Reference RNA (Stratagene) were direct labeled by random priming (Promega, Pronto). Spike-in controls were included together with the cell line hybridization mixtures. A 1 ng spike-in of 5' amino-modified 50-mer DNA oligos with sequence complementarity to either the sense or antisense strand of the β-lactamase fragment were incubated in 0.3 M hydroxylamine at room temperature with monofunctional Cynine5 or Cynine3 reactive dyes (Amersham). The labeled long-mers were mixed in each breast cell line targets (Cy3 label) or Human Reference RNA (Cy5 label). A similar labeling method was used for the M13 forward and M13 universal sequencing primers used to validate the single stranded nature of the array (Figure 2a–b). All hybridizations were performed in 4 × SSC, 0.1% SDS, with human 0.5 μg/μl Cot-1 DNA at 42°C overnight. Washes following hybridization were three times 4 × SSC RT, twice 2 × SSC/0.1% SDS at hybridization temperature, one time 0.2 × SSC and finally one time 0.1 × SSC RT. Slides were dried by centrifugation and scanned.

### Identification of potential non-specific cross-hybridization

Sequence information for the MGC clone inserts was retrieved from the MGC home page [16]. Repeat sequences were identified using RepeatMasker (A.F.A. Smit, R. Hubley & P. Green, unpublished data). Clone inserts with repeats for which the product of repeat length and similarity to the consensus sequence [1 – divergence] was greater than 40 were identified as repeat containing. Self-complementarity of strands was identified by aligning the reverse complement of the clone insert sequence against itself (NCBI blast; word size = 7, e-value cut-off = 1000). Sequences with self-complementary matches of 30 basepairs or more (> 79 % identity) were identified as self-complementary. Clone insert sequences and their reverse complement for all cDNAs were also aligned against the RefSeq mRNA database to identify sequences for which both the sense and antisense matched the same RefSeq mRNA over at least 30 basepairs with > 80 % identity (NCBI blast, word size = 7, e-value cut-off = 1000).

### Data analysis

Hybridized arrays were scanned using an Axon 4000A scanner (Axon Instruments). Acquired TIFF images were analyzed and individual spots were flagged as not found, found, or bad, in GenePix Pro 4 (Axon Instruments). The quantified data matrix was saved as a GenePix Results File (gpr) and loaded into a local installation of BioArray Software Environment (BASE) [17]. Subsequent pre-processing steps, within slide normalization, data filtration, and transformations were performed with in BASE. Median foreground pixel intensities for spots were adjusted by subtracting median background pixel intensities. Spots flagged as not found or bad during image analysis or considered saturated (containing more than 5% saturated pixels in either signal) were removed. Data within arrays were normalized to the median log2 ratio of sample intensity to reference intensity. Median log2 ratio was calculated using spots with both signal intensities above 100 and excluding the 5% highest and 5% lowest log2 ratios. Spots with both signal-to-noise levels below 10 were removed and replicated spots were merged. Hierarchical cluster analysis was performed using TMeV [18].

### Northern blot hybridization and RT-PCR validation experiments

For northern blots, 25 μg total Human Reference RNA (Stratagene) were loaded on 1,3% agarose gels containing formaldehyde. RNA Ladder High Range (Fermentas Life Sciences) was used as molecular weight standard. Electrophoresis was run in 1 × MOPS. The separated RNAs were transferred onto Hybond N+ nylon membrane by capillarity and subsequenlty cross-linked under UV light. P^32^-radiolabelled strand specific probes were synthesized by *in vitro *transcription (Riboprobe System, Promega). Hybridizations were performed in 50% (v/v) formamide, 5× SSPE, 5× Dendhardt's, 0,5% SDS, 100μg/ml boiled salmon sperm DNA at 42°C overnight. The blots were subsequently washed 3 times in 2 × SSC:0,1%SDS RT and twice 10 min in 0,1 × SSC:0,1%SDS at 65°C.

For RT-PCR, first strand cDNAs from each cell line were prepared from 500 ng total RNA. In all cases, reverse transcription was primed with a mixture of random hexamers using the Transcriptor First Strand cDNA Synthesis Kit (Roche Applied Sciences). One twentieth of the cDNA synthesis reaction was used as template in each PCR reaction. PCR reactions were primed with BC006233_for 5' TGTTTGTCAGCAAAGATGTGG and BC006233_rev 5' CTGGATGGAGGGGAGAAG. Expand High Fidelity polymerase (Roche Applied Sciences) was used for all reactions. Cycling conditions were: 2 min at 94°C, 20 seconds at 94°C, 30 seconds at 55°C, 2 minutes at 72°C and extension final of 10 minutes at 72°C. PCR products were resolved in 1% agarose gel electrophoresis and band intensities were measured with a Kodak 1D Image Analysis Software system.

## Authors' contributions

JVC performed the studies, microarray analysis and participated in the draft of the manuscript. JS constructed the microarrays and participated in the design of the study. AK and PM carried out the bioinformatic analysis and participated in revision of manuscript. ÅB participated in the design of the study. CR drafted the manuscript and conceived, designed and coordinated the study.

All authors read and approved the final manuscript.
